# Practicing virology: making and knowing a mid-twentieth century experiment with *Tobacco mosaic virus*

**DOI:** 10.1007/s40656-021-00481-9

**Published:** 2022-02-01

**Authors:** Karen-Beth G. Scholthof, Lorenzo J. Washington, April DeMell, Maria R. Mendoza, Will B. Cody

**Affiliations:** 1 Plant Pathology and Microbiology, Texas A&M University, College Station, TX 77843-2132 USA; 2 Present Address: Plant and Microbial Biology, University of California, Berkeley, CA USA; 3 Present Address: Plant Biology, University of California, Davis, CA USA; 4 Present Address: FujiFilm Diosynth Biotechnologies, College Station, TX USA; 5 Present Address: Chemical Engineering, Stanford University, Stanford, CA USA

**Keywords:** Science and experimentation, Tobacco mosaic virus, Plant genetics, Plant pathology, History of science, Complementary science, Reproducibility in science

## Abstract

*Tobacco mosaic virus* (TMV) has served as a model organism for pathbreaking work in plant pathology, virology, biochemistry and applied genetics for more than a century. We were intrigued by a photograph published in *Phytopathology* in 1934 showing that Tabasco pepper plants responded to TMV infection with localized necrotic lesions, followed by abscission of the inoculated leaves. This dramatic outcome of a biological response to infection observed by Francis O. Holmes, a virologist at the Rockefeller Institute for Medical Research, was used to score plants for resistance to TMV infection. Our objective was to gain a better understanding of early to mid-twentieth century ideas of genetic resistance to viruses in crop plants. We investigated Holmes’ observation as a practical exercise in reworking an experiment, having been inspired by Pamela Smith’s innovative Making and Knowing Project. We had a great deal of difficulty replicating Holmes’ experiment, finding that biological materials and experimental customs change over time, in ways that ideas do not. Using complementary tools plus careful study and interpretation of the original text and figures, we were able to rework, yet only partially replicate, this experiment. Reading peer-reviewed manuscripts that cited Holmes’ 1934 report provided an additional level of insight into the interpretation and replication of this work in the decades that followed. From this, we touch on how experimental reworking can inform our strategies to address the reproducibility “crisis” in twenty-first century science.

If a photograph is worth a thousand words, then we were taken (in) by an image from a 1934 scientific manuscript in the journal *Phytopathology* (Fig. 1). The figure shows a Tabasco pepper leaf dropping from the plant following inoculation with *Tobacco mosaic virus* (TMV). Tabasco plants respond to TMV infection within a few days of inoculation, first with localized necrotic lesions (LNLs) on the inoculated leaf. The LNLs are mere pinpoints, oftentimes all but obscured by the damage incurred by rub-inoculation. Leaf abscission occurs a few days after LNLs are observed. This response—to sacrifice an inoculated leaf to rid itself of the virus—is a dramatic outcome. Francis O. Holmes, a virologist at the Rockefeller Institute for Medical Research, used both responses to monitor for the presence of a dominant gene for resistance to TMV infection.^1^

**Fig. 1:**
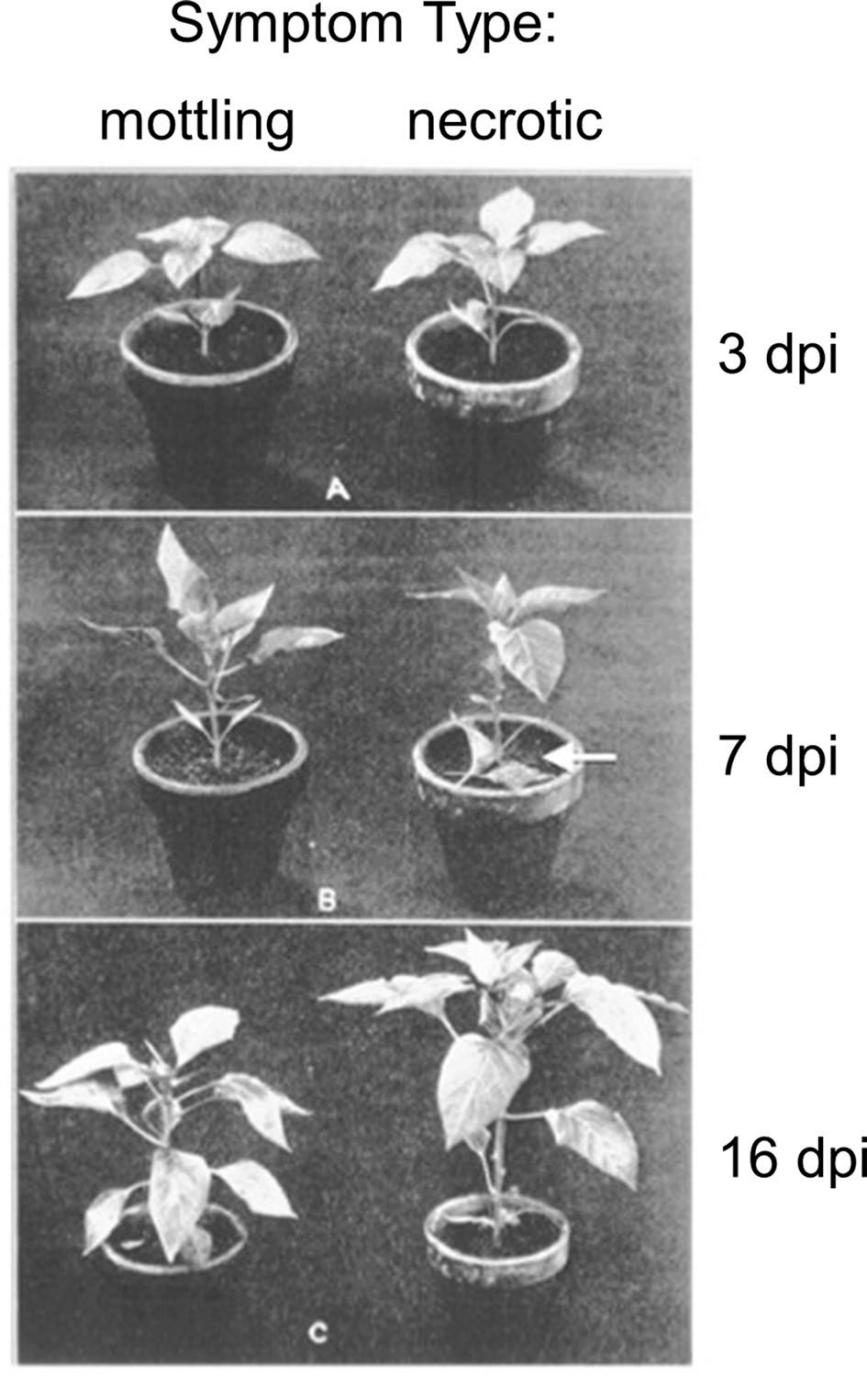
Photograph showing the effects of TMV-infection on homozygous (*ll*) and heterozygous (*LL* of *Ll)* plants from the genetic cross of Tabasco X bell pepper. The figure caption reads: “Two plants of *Capsicum frutescens*, inoculated with tobacco-mosaic virus. The first was a mottling-type plant and the second, a necrotic-type. A. 3 days after inoculation of 2 leaves each. B. 7 days after inoculation. Inoculated leaves had fallen from necrotic-type plant, freeing it from virus [arrow added for emphasis]. C. 16 days after inoculation. Mottling-type plant was stunted and mottled. Necrotic-type one was large, without symptoms, and free of virus” (Holmes, 1934, p. 988). The notations of symptom type, mottling (*ll*) and localized necrotic lesions (*Ll* and *LL* plants); and the days post-inoculation (dpi) with TMV were added to clarify Holmes’ experimental results. (Holmes, 1934, Fig. 2, p. 988, used with permission of the American Phytopathology Society.)

We were interested in replicating this experiment as an exemplar of “practicing virology” within the context of the history of science. Our work, initially inspired by Pamela Smith’s Making and Knowing Project, was fraught with challenges. A seemingly simple experiment belied the complexity and challenges of reworking an experiment from the past. We concluded that some experiments from the past cannot be replicated in full; that complementary methods are oftentimes necessary to interpret experimental results across the decades; that careful and attentive reading and interpretation of text and figures is necessary and essential to rework an experiment; and, identification and reading manuscripts that cite the original work is an extremely useful tool to interpretate historical experiments. Here we discuss our challenges and successes with reference to findings from historians of science who have reworked interesting experiments of the past. We also touch on the role of craft (making) and pitfalls associated with biological materials for historical reworking (knowing). In relation to a perceived “reproducibility crisis” in recent science, we discuss, in light of our experience, potential difficulties in reworking experiments, which includes identifying, replicating, funding and publishing the results. Finally, we hope our experiments will encourage more hands-on reworking as a key component of the historiography of the life sciences because of its informative value.

## Practicing virology

Prior to the rediscovery and wide-spread acceptance of Mendelian genetics, crop improvement was based on observation. Plant pathologists and breeders would survey fields, collecting seeds of plants with desirable traits, such as improved yield, or escape from the ravages of diseases. This seed would be increased and used in subsequent seasons. Another, more focused strategy, evaluated seed from local collections or that provided by the USDA.^2^ With Mendelian genetics, plant breeders in the twentieth century could deliberately introduce new, desirable traits to crop plants. Such “inheritable traits could be charted through mathematical probabilities” allowing for “efficient and predictable” outcomes including genetic resistance to plant pathogens (Campbell et al., 1999, p. 257). Seeds were harvested from plants with the desired phenotypes, followed by pathogen challenge of a new generation of (hybrid) plants. Plants tolerant or resistant to the challenge were advanced through the trials, grown to maturity and their seed harvested. Plants from these seeds, were backcrossed to plants with commercially desirable features. A stable genetic line would be developed with nearly all the original “good” features of a parent plant with the addition of genetic resistance to a particular pathogen. This work could take years. (While todays molecular methods allow for more rapid identification of the resistance genes, the breeding process remains labor and time intensive.) Finally, the seed would be increased for commercial use.^3^

**Fig. 2:**
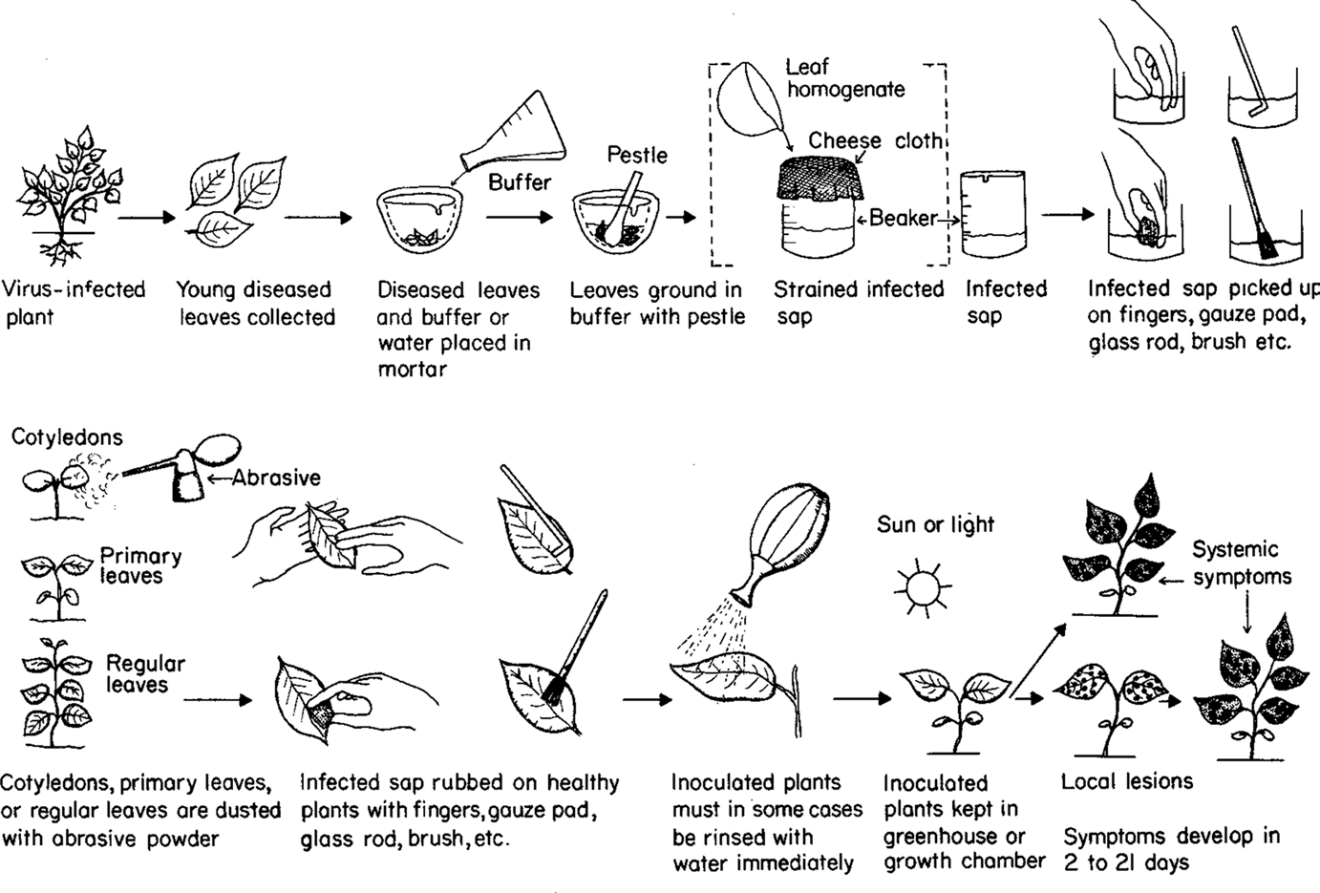
Illustration of mechanical inoculation of plant viruses as shown in the 2nd edition of *Plant Pathology*, a textbook by George N. Agrios (1978) used by generations of plant pathologists. (Agrios, 1978, Fig. 213, p. 568, used with permission of Elsevier.)

Tobacco mosaic virus was one pathogen causing economic losses in tobacco, pepper and tomato fields. In the early twentieth century, understanding the “nature” of the virus was an enormously difficult task as viruses could not be cultured or observed by light microscopy. By necessity, indirect methods were developed to study viruses and their interactions with host plants (Fig. 2). Francis O. Holmes was a scientist who is now recognized for creating innovative and reproducible advances in virology and plant breeding in the early twentieth century, first at the Boyce Thompson Institute for Plant Research (Yonkers, NY), then the Rockefeller Institute for Medical Research (Princeton, NJ). He reported on the development of a biological assay for plant viruses that involved the visualization of TMV infection on tobacco and other plants (Holmes, 1929b) (Fig. 2). Holmes observed small LNLs accumulating on TMV-inoculated *Nicotiana glutinosa* leaves. The virus was confined within the boundaries of the lesions on the inoculated leaf—this host response was protective, allowing the tobacco plant to complete its lifecycle without detriment. Holmes determined this response was due to *N. glutinosa* harboring a single dominant gene (*N*) for resistance to TMV infection (Holmes, 1929b, 1931; Scholthof, 2004, 2011, 2014). Then, he used Mendelian techniques to cross the *N. glutinosa* gene-*N* into *N. tabacum* (tobacco) as a first step to develop commercial tobacco lines with field resistance to TMV (Holmes, 1934, 1938; Scholthof, 2014, 2016).^4^ The LNL response to TMV infection was used as a biological assay to confirm the introgression of the *N*-gene into tobacco plants.

This process was fraught with difficulties in that it took Holmes three years to advance this project (Holmes, 1934, 1938; Scholthof, 2014, 2016). During this experimental interregnum Holmes pursued a similar approach with pepper (*Capsicum* species), finding it more amenable to Mendelian breeding strategies.^5^ He had determined that Tabasco pepper leaves inoculated with TMV developed small LNLs, then dropped from the plant a few days later (Fig. 1), rendering the plant virus free. Holmes attributed this effect to the presence of the Tabasco gene *L*, analogous to the *N. glutinosa N*-gene. With this knowledge, Holmes incorporated the Tabasco gene *L* into commercial lines of bell pepper, thus protecting the plants from systemic TMV infection.^6^ Today, this same *L*-gene is found in TMV-resistant bell pepper cultivars. The 1934 publication is important to plant pathology because it was the first demonstration that a resistance gene from one species could be used to protect another species from the ravages of virus infection. As shown by Holmes, Tabasco pepper leaves abscised within days of TMV inoculation, a striking means to visualize a gene-in-action in the pre-molecular biology era.

The manifest issues of technique, skill, tools, and temporal distance have been addressed by Pamela Smith’s pathbreaking “Making and Knowing Project” at Columbia University. Smith has commented on “how odd it is that historians whose object of study is historical materials and techniques … have generally not considered engagement with the materials of their historical topics as an essential part of their training and research” (Smith, 2016, p. 9). Here, acting as scientists and practitioner-historians, we investigate a historical topic and the value of tacit (or gestural) knowledge in experiment and interpretation. We concur with Smith that making and knowing is an “necessary part of our intellectual toolbox ... through hands-on work with materials and techniques,” and that the devil is in the details—some of which, as we will show, are details that we had initially not considered (Smith, 2016, p. 9). We explore, through demonstration, the complexity of “doing biology” across the decades. We found that although ideas travel, the biological components (plants, viruses) and performance of a technique are more difficult to locate.^7^

In his TEA set paper Harry Collins addressed the difficulty of replication across physical distance, even for those expert in their area of practice and craft (Collins, 1974).^8^ Collins interviewed TEA laser scientists, finding that peer-reviewed publications and citations were used to suggest “the flow of articulated and therefore visible information,” but this did not give a full understanding of “the modes of transfer of real, useable knowledge among a set of scientists” (Collins, 1974, pp. 170, 174). We are attempting to develop and construct parameters to transfer information—an experiment (Fig. 1). For Collins, the trial and error aspects of developing a new technology (tool) and “the non-systematic element” (Collins, 1974, p. 175) were part of the process of making and knowing, something we also encountered in setting up this “simple” TMV experiment.

Here, we provide an example of reproducing knowledge at a temporal distance, using a biological experiment. We encountered many of the same problems mentioned in the TEA set paper as we worked to reproduce an experiment from a written document. Collins indicated that “written sources … as the sole source of information” are inadequate and the ability to reproduce an experiment or build a piece of equipment or “reinvent it” indicates that the (naïve) group “knows as much” as the reporter (Collins, 1974, p. 176). Pamela Smith pulls these ideas together in a material framework (Smith, 2012). The “how to” comes about with deliberate reading, interpretation, testing, experimentation, and analysis of the results. In all instances repetition is key to mastering each step in the reworking—the craft of becoming a “maker”. Interpretation, analysis and extension of the findings is “knowing”. This iterative process entails significant time, material resources, hands-on experience, mistakes, troubleshooting, and critical thinking.

Several scholars have been at the forefront in engaging in the “experimental history of science” (Fors et al., 2016, p. 89) to deepen our understanding of the insight, craft, practice, and ideas of early physical and chemical scientists (Ahnfelt & Fors, 2016; Ahnfelt et al., 2020; Albala, 2010; Barwich & Rodriguez, 2020; Bilak, 2020; Chang, 2011; Fors et al., 2016; Hendriksen, 2020; Hendriksen & Verwaal, 2020; Principe, 1987; Root-Bernstein, 1983; Sibum, 1995; Smith, 2012; Usselman et al., 2005). Their contextualization of the historiography through experimentation brings us a richer understanding of scientific processes, development, and epistemology. Yet, little reworking has occurred within the life sciences.

One example of biological reworking was a counting study by Robert Root-Bernstein. This project revealed the difficulty of reproducing a seemingly straightforward problem in biology: identification by observation of seed characteristics (the phenotype) using maize kernels (Root-Bernstein, 1983). As described by Root-Bernstein, the early twentieth century controversy surrounding the results of Mendel’s garden pea study (when 1936 Ronald Fisher proclaimed that the counting must be off or that some fudging occurred because it surely was not possible to have those precise predicted ratios) could be resolved by a simple experiment. Instead of using peas, Root-Bernstein selected maize, using a monohybrid cross (pure lines of purple seed X yellow seed parents); the hybrid would produce, according to Mendel, an equal ratio of purple:yellow kernels. He asked undergraduate students to count the number of purple or yellow kernels on an ear. Root-Bernstein found that it is more difficult to assess a phenotype (the physical expression of a gene) than expected, with upwards of 2% of the kernels “indeterminant” or “difficult to classify”. However, the general results were in line with what was predicted by Mendelian ratios. A more difficult task of scoring two dihybrid crosses with the “traits purple, yellow, wrinkled and smooth,” classified 6% of the kernels as “indeterminate” (Root-Bernstein, 1983, p. 284).

Root-Bernstein’s work leads us to a similar experiment reported by Raymond Pearl in 1911 (Pearl, 1911). Pearl used “fifteen trained observers” who “were required to discriminate only with reference to the color [yellow or white] and the form [starchy (smooth) or sweet (wrinkled)] of each kernel” with the expected Mendelian second generation ratios of 9 yellow starchy:3 yellow sweet:3 white starchy:1 white sweet (Pearl, 1930, p. 127).^9^ All observers counted 532 kernels, yet none of the “highly trained and competent observers” were in agreement concerning the distribution of the characteristics (Pearl, 1930, p. 129). Pearl wrote that this “seems a simple problem. One only has to count them. They [the kernels] do not run away or change” (Pearl, 1930, p. 129). This was a reworking at the most simple state – no preparation of plants, chemicals, inoculation, or cultivation. Merely counting. Root-Bernstein found that with practice, the students became better at making choices and in which bin to place the kernels. This outcome reminds us of the comment by Barbara McClintock to Evelyn Fox Keller that there is “a feeling for the organism” or, something that develops over time, allowing the experimentalist to ‘see’ and ‘understand’ more deeply with immersion than as a novice (Keller, 1984). We suggest that this is the beauty of reworking experiments. Making and knowing allows us to understand more about the methods and conclusions reported on by historical actors, the constraints associated with materials available in a given time period, and the experiential skills needed to accomplish fundamental, interesting studies in the sciences. The TMV-pepper experiment seemed an ideal project to learn more about Holmes’ ideas and his standard practices.

## Making: materials and methods

The impetus for this experiment was the dramatic image of pepper leaf abscission several days following the LNL response to TMV inoculation, as shown by Holmes (Holmes, 1934) (Fig. 1). Our objective was to gain a better understanding of early to mid-twentieth century ideas of genetic resistance to viruses in crop plants.^10^ Identifying the working biological materials (TMV strains and pepper plants) used by Holmes for his experiments was non-trivial. With only the briefest textual description of his methods in his published scientific papers, we had to interpret the experimental design.

### Plants and planting

We purchased Tabasco and Heirloom California Wonder (sweet bell pepper) seeds from W. Atlee Burpee & Co. Two TMV-susceptible tobacco lines, *N. tabacum* cv. Turk and *N. benthamiana* (commonly used for laboratory experiments), were cultivated from our laboratory seed stock.^11^ All plants were grown using the conditions shown in Table 1.^12^

**Table 1:** Known variables based on Holmes’ pepper experiment

Variable		Holmes (1934)	Reworked
*Viruses*	TMV	field type TMV James Johnson ?^a^	Probably U1 strain^b^
	TMV-GFP	n/a	Lindbo (2007)
*Plants*	Tabasco	Walter Greenleaf ?	W. Atlee Burpee Co.^c^
	bell pepper	Campbell Soup Co. ?	W. Atlee Burpee Co.
*Virus maintenance*		*Nicotiana glutinosa, Nicotiana tabacum*	*Nicotiana benthamiana, Nicotiana tabacum* (Turk)
*Plant growth conditions*	lighting	greenhouse^d^	growth chamber (125 µE); light bench (150 µE)
	day/night	?	16 h light/8 h dark
	temperature	25 °C	22–25 °C (growth chamber); 20–22 °C (light bench)
	soil	?	Promix^e^
	pots	4″ clay	4″ plastic
	fertilizer	?	20–20-20^f^
*Plant age*		? (4 leaves)^g^	8 week old plants (3 leaves, similar size)
*Inoculation*		water ?	50 mM KH_2_PO_4_
*Abrasive*		?	1% Celite^h^
*Rinse leaves*		yes	yes

^a^A question mark (?) indicates a best guess of the source based on manuscripts, reports and correspondence

^b^TMV U1 strain is available from the American Type Culture Collection (https://www.atcc.org), catalog number PV-135

^c^Tabasco (22,661) and bell pepper seeds California Wonder (60816A), Bullnose (64495A), Chinese Giant (51888A) were sourced from W. Atlee Burpee Co., 2018–2019 catalogs (www.burpee.com)

^d^Also “outdoor conditions in the [Boyce Thompson] Institute gardens” (Holmes, 1932, p. 323)

^e^Promix (PBPGX28) growing medium with sphagnum peat moss (75–85%), vermiculite, limestone, and wetting agent was sourced from Premier Tech Horticulture (www.pthorticulture.com)

^f^General purpose 20–20-20 (N-P-K) fertilizer from J. R. Peters (G99290; www.jrpeters.com). Plants were treated weekly with 0.25 ppm fertilizer in water

^g^Plant age for Holmes’ experiment was determined from the photograph shown in Fig. 1, adapted from Holmes (1934)

^h^Celite is powdered diatomaceous earth used as an abrasive to damage the leaf, allowing virus entry into the cells

Commercially produced seed introduces additional genetic variables, although the plants may appear to be identical (phenotype). For example, a genetic analysis of ten lines of California Wonder showed the plants could be grouped into 5 classes, based on genetic polymorphisms identified by PCR amplification with a series of primer sets to randomly sample the genome (Votava & Bosland, 2002). The authors cautioned that California Wonder “exists in name only” and its utility as a standard control should be determined based on the type of experiments performed (Votava & Bosland, 2002, p. 1101).^13^ Similarly, for Tabasco (*C. frutescens*) it is not possible to definitively state that the plant is identical to Holmes’ Tabasco; almost certainly it is not the same.^14^ However, from observations made by Walter H. Greenleaf, a plant breeder and pathologist at Auburn University (Alabama), we know that “the *L*-gene in peppers provides an effective form of resistance” to “all tested strains of TMV from tobacco and tomato” (W. H. Greenleaf, 1986a, 1986b, p. 98), giving us a degree of confidence that a commercially available of Tabasco would be suitable for these experiments.

### Rub inoculation

In the late 1920s, in a series of experiments, Holmes developed the rapid and efficient inoculation technique, now a standard practice, known as mechanical (or rub) inoculation (Holmes, 1928, 1929a, 1929b, 1931) (Fig. 2). For rub-inoculation, one or more TMV-infected symptomatic *N. tabacum* leaves were pulverized with the addition of water or phosphate buffer (1:10 w/v), using a mortar and pestle. The negative control experiment, or mock-inoculation utilizes healthy leaves. Two or three lower leaves of a plant are rubbed gently with the sap extract following dusting with an abrasive powder (carborundum or Celite) to slightly injury the leaf, allowing virus ingress (Kalmus & Kassanis, 1945). Immediately after inoculation the leaves were rinsed with water. The plants were observed every day and symptoms were recorded, with particular attention to local lesions and systemic infections.^15^

### Tobacco mosaic virus (TMV)

**Fig. 3:**
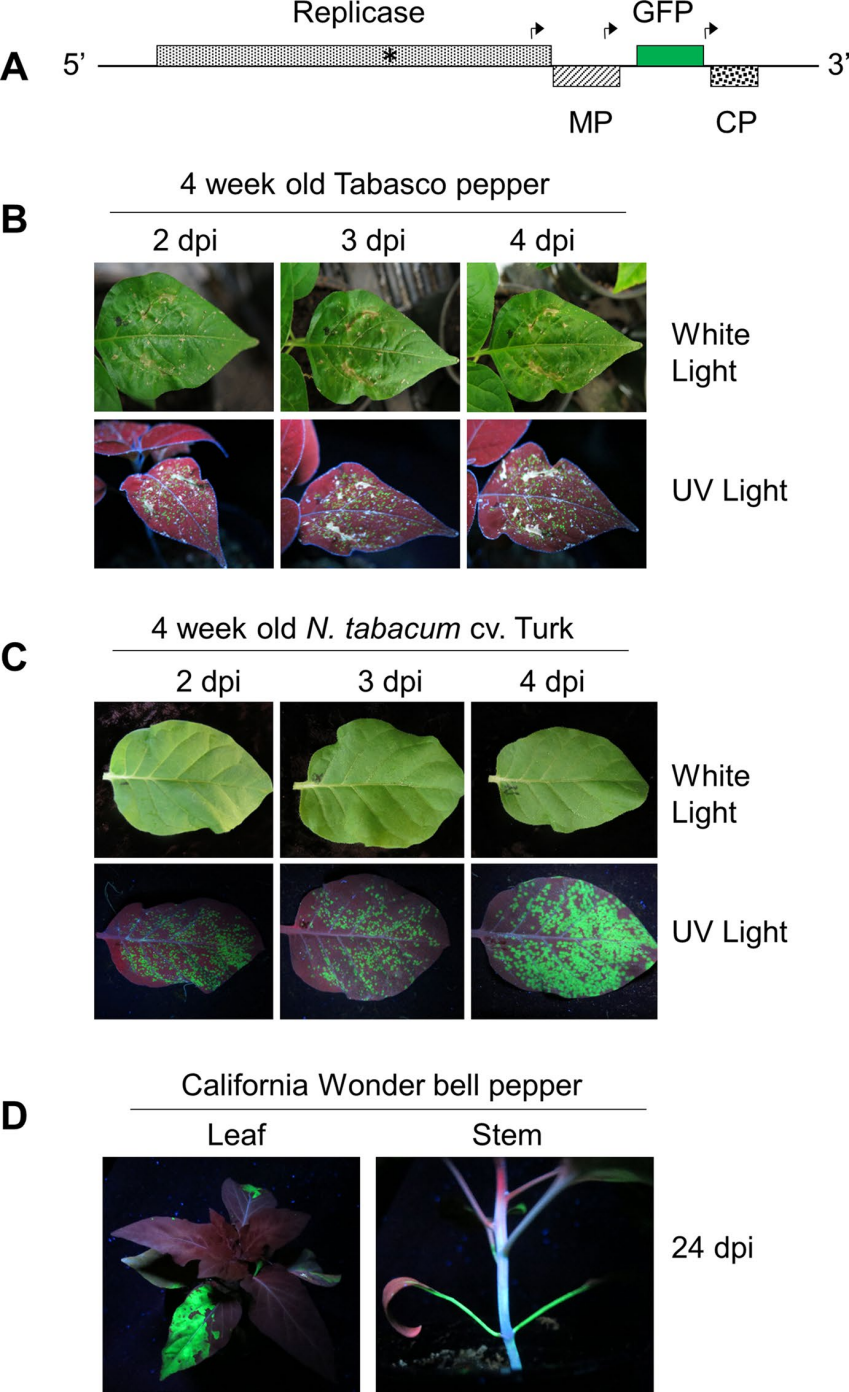
Exploring Holmes’ results with *Tobacco mosaic virus* (TMV) and pepper plants with the techniques of molecular biology. **a** The molecular genetic map of TMV with the addition of a reporter gene encoding the green fluorescent protein (GFP). The rectangles indicate protein-encoding genes of TMV: replicase, movement protein (MP), and capsid protein (CP). The bent arrows indicate the subgenomic RNA promoters. The asterisk indicates that a specialized strategy of readthrough translation to express two replicase proteins from the genomic RNA. **b** and **c** Representative Tabasco pepper (*Capsicum frutescens*) and *Nicotiana tabacum* cv. Turk (tobacco) leaves at 2, 3 and 4 days post-inoculation with TMV-GFP. The leaves were photographed under white light and ultraviolet (UV) light. In Fig. 3B, rub-inoculation damage of the inoculated leaves presents as brown discoloration under white light and greyish-white discoloration under UV light. The same leaves were used for white and UV light exposure. The localized green fluorescent spots on Tabasco and tobacco leaves reflect single infection events following inoculation with TMV-GPF, equivalent to the localized necrotic lesions reported by Holmes. On tobacco, the pinpoint florescence spots at 2 dpi become much larger by 4 dpi, indicating TMV resistance gene *N* is not present. In time these green fluorescent spots coalesce and progress to systemic infection (not shown). D. California Wonder bell pepper (*C. annuum*) plants showing systemic infection at 24 days post-inoculation with TMV-GFP

The TMV common strain (U1) was maintained on *N. tabacum* cv. Turk and *N. benthamiana*. This strain induces necrotic local lesions on *N. glutinosa* and Tabasco pepper. Pepper leaves were rub-inoculated following Holmes’ method (Fig. 2). Unfortunately, due to rub inoculation damage on our plants, it was difficult to count lesions and to determine the level of infection. To rework this experiment we used a more tractable tool, an infectious TMV cDNA construct. This is a routine plant molecular virology practice to determine if pepper plants were susceptible to TMV infection.^16^ Specifically, our complementary experiment utilized a molecular construct of TMV with the addition of the green fluorescent protein (*gfp*) gene (TMV-GFP) (Fig. 3A). TMV-GFP infected tobacco leaves were harvested and used as inoculum for the pepper experiments (Fig. 2).^17^ TMV-GFP was used to i) monitor virus infection (count local lesions) by fluorescence under ultraviolet light and ii) determine the sites of virus replication versus inoculation damage. TMV-GFP provided consistent and genetically homogeneous inoculum to investigate TMV infection, development of LNLs, and leaf abscission.

Other historians of science who had pursued their own reworking of experiments, reported using modern tools as they developed their craft (Ahnfelt & Fors, 2016; Ahnfelt et al., 2020; Albala, 2010; Barwich & Rodriguez, 2020; Bilak, 2020; Chang, 2011; Fors et al., 2016; Hendriksen & Verwaal, 2020; Principe, 1987; Root-Bernstein, 1983; Sibum, 1995; Usselman et al., 2005). For example, Hasok Chang uses modern instruments to understand historical experiments. For his “complementary” experiments on the boiling point of water, he explained “when practitioners of historical replication say they try to get ‘as close to the original as possible’, that is usually with a clear awareness of some inherent limits to faithfulness. It is not always possible to match exactly the past instruments and operations described in historical papers” (Chang, 2011, p. 320). Chang also notes the historic manuscript may exclude some methodology because it was “well-understood by readers in the original context” (Chang, 2011, p. 320). For these reasons, we introduced complementary molecular virology tools as “*opportunities* for better historical understanding” (Chang, 2011, p. 321).

## Knowing: results

We rub-inoculated two or three leaves of small Tabasco and bell pepper plants with sap of TMV or mock-inoculated plants for the control experiment. Our expectation was that visible chlorotic lesions would develop on California Wonder bell pepper leaves within 7 to 10 days, followed by mottling of the upper, non-inoculated leaves. For Tabasco pepper, we expected to observe LNLs within a few days, followed by leaf abscission. Instead, in our hands, abscission was observed for mock- and TMV-inoculated bell pepper and Tabasco plants.

We were especially confounded by the abscission response we observed on mock-inoculated leaves. Holmes consistently emphasized that rapid leaf drop was a marker for the Tabasco *L*-gene. For example, TMV-infected *L*-gene segregating bell pepper lines, such as California Wonder, “show necrotic primary lesions only, and their inoculated leaves were soon lost by abscission” (Holmes, 1937, p. 641).^18^ However, Holmes also reported that older plants as well as plants maintained under different environmental conditions may defoliate independent of *L*-gene-associated abscission (Holmes, 1932, p. 352). Altogether, we decided to focus our attention on (1) environmental conditions; (2) inoculation techniques, (3) confirming the mock-inoculated plants were not contaminated with TMV; and (4) the possibility of genetic variability of California Wonder, such as the inclusion of the *L*-gene or minor resistance genes or inadvertent contamination of seed lots.

As we know from Holmes, California Wonder is susceptible to TMV infection (Table 2).^19^ Yet genetic variability within California Wonder occurs, as reported by Eric Votova and Paul Bosland, pepper breeders at New Mexico State University. This variability is a result of inadvertent mixing of seed lots by producers, intentional selection by plant breeders over time, or genetic drift (Votava & Bosland, 2002). Of course, it is impossible to rework the experiment with the exact same seeds Holmes used, which may have affected our interpretation of his findings (Table 2). However, we did determine that California Wonder was susceptible to TMV (Fig. 3D). We then narrowed our considerations to environmental conditions and plant age.

**Table 2:** Summary of effects of tobacco mosaic virus infection on *Capsicum* (pepper) plants (Holmes, 1937)

Response	Inoculated leaf	Non-inoculated leaf (systemic infection)	Pepper variety	Gene^a^
Systemic chlorosis type	Yellow primary lesions	Mottled, distorted leaves	Bell pepper, pimiento pepper^b^	*ll*
Localized necrosis type	Localized necrosis (2–3 dpi); abscission (> 4 dpi)^c^	None	Tabasco	*LL*
Delayed-necrosis type	Symptomless	Few, relatively inconspicuous, yellowish lesions; delayed necrotic lesions on young emerging leaves, abscission	Long Red Cayenne, Sunnybrook, Ruby King	*l*^*i*^*l*^*i*^

^a^Genetic resistance to TMV infection was ranked by Holmes using the notation *LL* > *l*^*i*^*l*^*i*^ > *ll* (Holmes, 1937)

^b^Commercial bell pepper, such as California Wonder, was described as large fruited, blocky shaped, non-pungent. Plants with the *l*^*i*^*l*^*i*^ background had “imperfect localization” of TMV, showing “delayed necrosis with leaf abscission” (Holmes, 1937, p. 642). The *l*^*i*^*l*^*i*^ allele was associated with some commercial bell pepper varieties, including Ruby King. In the field, TMV-infected pepper plants with the *ll* allele were mottled and stunted, with significant reduction in yield and quality

^c^Days post-inoculation with TMV (dpi)

Early on we observed leaf drop in almost all peppers—this was particularly evident when there were changes in the environment, including decreased temperatures in the growth chamber, or lab, due to power failures or maintenance issues, or biological contamination of the growth chambers with insects and fungi (a complication of working in shared spaces in a plant pathology department). Our first estimation of plant age was based on plant height and the approximate leaf size (Fig. 1; 4-inch diameter clay pots). We returned to Fig. 1 and determined that Holmes had used more mature plants, based on a count of the visible internodes. From this, we decided it would be worthwhile to test older plants for the abscission response.

## Doing it again: laboratory practice and practicing

Plant virus inoculation and the molecular biology technique of the plasmid prep (isolating plasmid DNA from bacteria, generally *E. coli*), are both considered straightforward “ubiquitous practice” (Jordan & Lynch, 1992, p. 78). These methods of practice are so basic that they are used in undergraduate laboratory exercises (Dijkstra & De Jager, 1998; Ford & Evans, 2003). As elaborated by Kathleen Jordan and Michael Lynch, seemingly rote processes are predicated on more than the ability to read a protocol. Oftentimes there are “persistent problems associated with establishing the coherence and efficacy of the practice, determining whether one practitioner’s method for doing it is the same as another’s, accounting for discrepant results, and explaining how the technique works” (Jordan & Lynch, 1992, p. 77). Importantly, this is in spite of the protocol being “relatively standardized, reproducible, coherent, and subject to rational reconstruction” (Jordan & Lynch, 1992, p. 77). Yet protocols are neither rational or standardized without technique—typically acquired through apprenticeship. Here we are evaluating two aspects of a “mundane practice” (Jordan & Lynch, 1992, p. 78): i) are Holmes’ observations reproducible in our hands? And ii) what sort of expertise matters to recapitulate previously published data?

In Jordan and Lynch’s study, they interrogated practitioners to learn about differences in a common practice, asking about variation “between their own and others’ methods” as well as “local circumstances of the lab and idiosyncrasies of its members” (Jordan & Lynch, 1992, p. 78). Like the plasmid prep, virus inoculation is a key, mundane practice that must be learned (Figs. 1, 2 and 3). Pamela Smith and Tonny Beentjes discuss this “makers’ knowledge” within the context of reconstructing life-casting techniques in the sixteenth-century. They emphasized that the “knowledge possessed by handworkers, also known as ‘makers’ knowledge’” is key to understanding the materials, techniques and “how and why nature was investigated” (Smith & Beentjes, 2010, p. 130).

The “simplicity” of TMV inoculation of tobacco is made evident by its common use as an experiential tool for in plant pathology laboratory courses (Dijkstra & De Jager, 1998; Ford & Evans, 2003). Yet, rub inoculation is a particular practice subject to many errors, including damaging plants by rubbing leaves with too much enthusiasm (Fig. 3A). The experimental outcome “can depend on the particular ingredients used, as well as an endless array of other circumstantial features” (Jordan & Lynch, 1992, p. 81), even for a virus inoculation method standardized in the mid-1930s.^20^

**Fig. 4:**
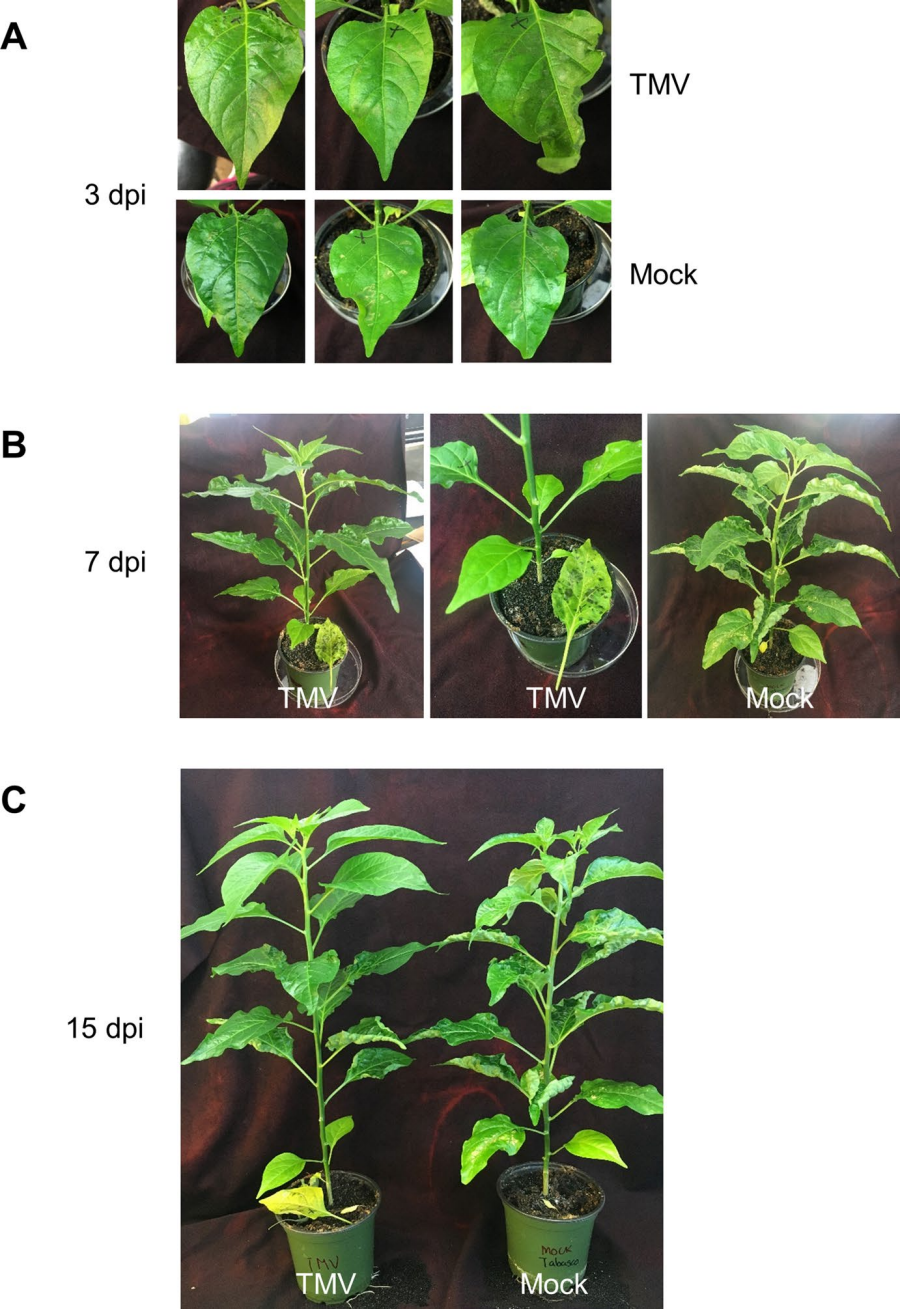
Recapitulation of the Tabasco pepper experiments described by Holmes. Panels **a**, **b**, and **c**. Wildtype TMV inoculated to Tabasco, as shown in Fig. 1, and photographed at 3, 7 and 15 days postinoculation (dpi). On Tabasco leaves the necrotic pinpoint local lesions are difficult to observe, especially when the leaves are damaged during inoculation. **b** and **c** The TMV-inoculated leaf abscission noted at 7- and 15-dpi on two plants; mock inoculated leaves at 7 dpi have not abscised. An “X” on the leaf indicates that the leaf was inoculated (TMV or mock). In B, the center figure is a close up of the dropped leaf shown in the leftmost photograph. These results can be compared to those Holmes (1934), shown in Fig. 1

We systemically compared our materials and methods to those reported by Holmes (Table 1) and identified many variables, some of which may have affected the outcome of our reworking experiments. For example, in our hands pepper was exquisitely sensitive to environmental conditions, especially changes in ambient temperature. When we returned to the text, making a more careful study of his publications we found that Holmes had reported that TMV-susceptible *Capsicum* (and several other plant species) exposed to cooler growing temperatures may experience premature leaf abscission (Holmes, 1932, p. 337). Another identified variable was plant age. When carefully inspecting Fig. 1, we noticed that Holmes’ plants had several internodes, indicating more mature plants. In our subsequent reworking experiments, we used older plants. But our plant growth conditions resulted in tall plants with with elongated internodes, a result of low light intensity (Fig. 4). The variables that had foiled our initial efforts encompassed the key determinants of infection: the host, the virus, and the environment. Parsing the most important variables towards becoming proficient with Holmes’ methods, we realized the experimental protocol had features that were strikingly similar to those mentioned by Jordan and Lynch: “Although the plasmid prep is far from controversial and is commonly referenced as a well-established and indispensable technique, how exactly it is *done* is not effectively communicated, either by print, word of mouth or demonstration. Instead, it is mastered largely through repeated (and often solitary) practice” (Jordan & Lynch, 1992, p. 84).

Initially this seemed a straightforward project to gain some understanding about how Holmes performed his experiments and if we could achieve similar results. What we know now is that these seemingly trivial experiments were fraught with technical difficulty and a great deal of complexity, even though we were merely pulverizing a TMV-infected leaf, rubbing it on a healthy pepper leaf and observing the outcomes of infection (mottling, leaf drop, etc.). The choices we and Holmes made were not trivial or insignificant. As discussed by Jordan and Lynch in their analysis of the standardized protocol for plasmid preps: “For practitioners at the bench, these distinctions [choices] do not easy resolve such issues as what to include or exclude from a procedure, what to do now, and what to do next” (Jordan & Lynch, 1992, p. 100). We were handicapped by the lack of detailed protocols and an ever increasing number of parameters to attend to. Upon reflection, the lack of detailed materials and methods is not a Holmes-specific issue, nor is it an issue limited to historical biological reworking.

## Reading between the lines: reproducibility

Our difficulty in reworking a decades-old experiment gets at broader questions. Is it possible to reproduce an experiment? Is an experiment valid if it never is reproduced? Perhaps the reproducibility is what makes co-discoveries from different labs so exciting—the realization that a phenomenon is “real” and others notice it as well. This suggests that there is merit to multiple groups tackling similar questions. These issues are of considerable interest as the science community has faced concerns about the reproducibility of peer-reviewed data and, in extensively corrected or retracted papers, if the remaining data has value in a given manuscript.^21^

The early outcomes of our experimental reworking were both frustrating and dissatisfying. We had success in observing localized necrotic lesions and abscission, but wondered if abscission was the gold-standard bioassay to score peppers for TMV resistance. Did contemporaries of Holmes confirm his findings or report useful modifications that we were unaware of when we initiated our experimental reworking? Is this method used today to evaluate TMV resistance in *Capsicum*?

This sort of closer reading and (re-) interpretation is integral to science practice. For example, Staffan Müller-Wille and Giuditta Parolini inspected copies of Mendel’s pea breeding manuscript, finding that readers “actively engaged with the text” by “rehearsing calculations and by employing Mendel’s notation system” (Müller-Wille & Parolini, 2020, p. 157). The annotations and underlining revealed what the reader “deemed most important” (Müller-Wille & Parolini, 2020, p. 153). Today, genetics students learn to use Punnett’s square for visualizing the outcome of genetic crosses of dominant and recessive genes. This “interplay between text and image” (Müller-Wille & Parolini, 2020, p. 163) and annotation, for us, was an important part of a process that revealed Holmes’ ideas, to identify experimental materials and methods, and to design complementary experiments. Later, we repeated this process as a tool to troubleshoot possible errors in reworking the pepper experiment. As Müller-Wille and Parolini write, this “active engagement” is a fundamental aspect of research “alongside the observations conducted at the lab bench and the experimental garden” to interpret and gain “practical knowledge” (Müller-Wille & Parolini, 2020, pp. 164, 165). Similarly, Pamela Smith showed us the importance engaging with material objects, text and drawings, to recreate skills and knowledge of the past (Smith, 2012). Nils-Otto Ahnfelt and Hjalmar Fors also reported that they “should have returned to the sources and read the original recipes more carefully”; consulted other sources to inform a practice or provide an “indirect pointer” for troubleshooting and problem-solving; and, performed “complementary” experiments with modern instrumentation to replicate the historical work (Ahnfelt & Fors, 2016, p. 177).

To evaluate the reproducibility of Holmes’ experiment in others’ hands, across a gap of nine decades, we identified 17 manuscripts, from 1936 to 2021, that reported on TMV inoculation of *L*-gene pepper plants and also cited Holmes’, 1934 or 1937 *Phytopathology* papers (Table 3). Of these papers, all 17 reported LNLs following inoculation of *L*-gene pepper plants with TMV; 12 also reported leaf abscission. Two authors reported they obtained *L*-gene bell pepper seed from Holmes (Greenleaf, 1953; Murakishi, 1960). Harry Murakishi, at Michigan State University, used Holmes’ “LL-resistant garden pepper”, reporting LNLs appeared following TMV inoculation, but he did not indicate leaf abscission (Murakishi, 1960, p. 464). Walter H. Greenleaf, a pepper breeder at Auburn University, regularly exchanged seed and viruses with Holmes, and on several occasions reported that Holmes’ *LL*-bell pepper responded to TMV inoculation with LNLs and abscission (Greenleaf, 1953, 1986a, 1986b). Greenleaf also used Holmes’ *L*-gene bell pepper line to develop a TMV-resistant pimiento pepper (Greenleaf et al., 1969).

**Table 3:** Citation analysis of selected manuscripts published by F. O. Holmes (1929a, 1929b, 1934, 1937, 1938)^a^

Year^b^	Plant/Experiment^c^	10 years (self-cites)^d^	Total citations^e^	Journal
1929	tobacco/LNLs	38 (2)	172	Botanical Gazette
1934	pepper/LNLs/abscise	7 (5)^f^	51	Phytopathology
1937	pepper/*L*-gene	2 (2)	47	Phytopathology
1938	tobacco/*N*-gene	20 (1)	214	Phytopathology

^a^Analysis performed using Web of Science (https://clarivate.com) with search parameters “Holmes FO (Author) and 1929–1938 (Year Published)”. The number of peer-reviewed articles citing the original publications are shown with citation data. Four journal articles were used for the citation analyses (Holmes, 1929a, 1929b, 1934, 1937, 1938)

^b^Year of publication of the four papers used by Holmes used for citation analysis (Holmes, 1929a, 1929b, 1934, 1937, 1938)

^c^Tobacco indicates *Nicotiana* species, pepper indicates *Capsicum* species; LNLs refers to localized necrotic lesions on an inoculated leaf following TMV infection; abscise indicates the inoculated leaf drops from the plant following TMV infection; and, *L*-gene and *N*-gene refer to dominant resistance gene in Tabasco pepper and *Nicotiana glutinosa*, respectively, introgressed into crop plant varieties

^d^Total citations of the paper in the decade following publication. The parentheses indicate the number of self-citations (self-cites) by Holmes in subsequent publications for that decade (a subset of total citations)

^e^Total citations from year of publication through October, 2021

^f^Both papers citing Holmes were on the general topic plant (pepper) breeding, not the experimental use of TMV

H. H. McKinney, a USDA plant virologist, found that TMV-inoculated *Capsicum frutescens* (*L*-gene) plants maintained at 23ºC developed “local necrotic lesions on mature and nearly mature leaves … and these leaves eventually absciss” (McKinney, 1937, p. 55). Similarly, Glenn S. Pound and G. P. Singh at the University of Wisconsin, noted “necrotic lesions on inoculated leaves. At all temperatures, inoculated leaves abscised and the plants remained free of systemic infection” (Pound & Singh, 1960, p. 805). In 1968, Mo-Yeong Lee and Paul G. Smith at the University of California-Davis, scored pepper lines for TMV-resistance by the LNLs response “just before leaf abscission” (Lee & Smith, 1968, p. 1445). But twelve papers, most of which did not cite Holmes' TMV-pepper work, did not mention abscised leaves in response to TMV infection, suggesting that it was not a consistently reliable assay (as we found) or it added no additional value to the standard scoring for LNLs.

Donna Bilak and her colleagues in the Making and Knowing Project, remind us that “recipe literature is a challenging genre to read, not only because of its frequent technical obscurity and abridged prose, but often even more so because of its simple style and apparent straightforwardness” (Bilak et al., 2016, p. 41). The same can be said the materials and methods sections of peer-reviewed scientific manuscripts and protocol manuals.^22^ Ken Albala, a culinary historian and practitioner, reminds us of the importance of Renaissance cooks who “recorded their extensive experience” even if the methods are unfamiliar to us; in short, “we must trust what is on the page” (Albala, 2010, p. 87). Similarly, the experimental work of Lawrence Principe reveals that reconstruction of the alchemy is possible because the work is “grounded in chemical reality, even though a simple reading of the text by a person well-versed in chemistry might well suggest the contrary” (Principe, 1987, p. 27). Yet, peer-reviewed manuscripts often have insufficient details to reproduce experiment. And becoming adept with new techniques and tools, such as construction of a TEA laser (Collins, 1974), may require communication with the innovators, spending many frustrating weeks to years to become expert in the method, or waiting for a commercial company to develop a kit, machine, or service to standardize the technique.

In our pursuit of a decades old experiment using established, standard methodology we found nearly every element (soil, watering regimen, plants, lighting, inoculum, and pest control measures) affected our ability to make and learn from Holmes. This has ramifications for scientists and historians who decide to replicate key experiments in their field. As we show here, and as discussed by Jordan and Lynch for plasmid preps, success with a particular protocol belies the depth of required experience and expertise by the users. Our difficulty in recapitulating Holmes’ work was difficult and interesting, and we learned a few things:

1. We became better readers and observers. As noted by Pamela Smith and others, we too stumbled on the processual research, providing us with the opportunity (and necessity) of studying processes rather than discrete events, to carefully read the materials and methods, and then assemble the needed reagents and tools. This extends into the need for carefully reading the protocols following failed attempts. In the early stages, the process of re-investigation seemed straightforward. Yet it quickly became evident that we were missing or unable to identify the materials used by Holmes. We interpreted what we expected to observe; that is, we anticipated ‘seeing’ the exact outcomes shown in Fig. 1, but we had not realized that a particular combination of plant age, lighting and temperature would determine the outcome. We did not review the literature citing Holmes, as we were intent on reworking his experiment. However, this literature revealed that LNLs were sufficient to identify TMV-resistance plants. Leaf abscission offered no added value when studying *L*-gene pepper plants or developing new commercial varieties.

2. Side projects are projects. The TMV-pepper experiment was piggy-backed onto ‘normal’ experiments in our molecular virology laboratory. We do not often perform experiments unrelated to our primary research interests. “What is typical, rather, are extended series of experiments which communicate among each other with different intensity and constitute an experimental texture,” as noted by Hans-Jorg Rheinberger (Rheinberger, 2001, p. 53). The TMV experiment dislocated us from our normal science practice, re-enforcing that that tacit knowledge and its implicit peculiarities are relevant to the success of the practitioner (Keller, 1984). We greatly underestimated the time and effort required to rework this experiment, because we were confident that the experiments were simple, and could be managed as a ‘side project’. Instead, we found that it takes time, money, and intensive focus to rework an experiment—there are no shortcuts.

Repeating this ‘simple’ experiment cast doubt on our expertise, leading us to revert to our familiarity with recombinant DNA tools to visualize the results. In Jordan and Lynch’s study of the plasmid prep, interviews with practitioners made evident that there is both a “black box” aspect and a “reflective” aspect to this work, and an individual in a laboratory (and an individual laboratory), may have strong feelings “over just what sorts of variations are tolerable, trivial, or significant” (Jordan & Lynch, 1992, p. 105). It is inevitable that we work with available materials and these may change over time (plant lines, virus strains), specific conditions (laboratory infrastructure) and expertise (Holmes’ experiences versus our experiences). From the outlines and framework presented by Holmes, we have re-realized the complexity of our ‘everyday’ work in the laboratory. The devil *is* in the details.

3. Reproducibility is experimentation. We were humbled by the complexity of repeating an experiment from 1934. What we found, reiterating the analysis by other re-workers in the making and knowing community, is that written materials and methods are important, but are not technical guides or how-to manuals to replicate an experiment. The repetition, frustration, and mastery of techniques are part and parcel of doing science. Of equal importance are the ineffable influences of mentors and peers, institutions, classroom knowledge and laboratory training and a dash of serendipity that affect the successes, failures, interpretation and presentation of data.

The difficulty of repeating this work speaks to a larger issue in the biological science—reproducibility. Reproducibility in science has relevance to scientists, historians, philosophers, publishers and funding agencies. Journals provide guidelines to authors, emphasizing that the materials and methods should be sufficiently detailed for the work to repeated or replicated.^23^ Yet, from our ‘simple’ experimental reworking, the written manuscript was not sufficient—crucial information was found in a photograph (Fig. 1). Perhaps this not surprising, because images (drawings, photographs, video, and models) “are especially effective in organizing technical knowledge into an abbreviated form” (Smith, 2012, p. 24).

Pamela Smith and Hasok Chang have shown us through their historiographic reconstructions that the text and even drawings are not enough; making is process of “observation and imitation” of experts, oftentimes requiring complementary experiments (Chang, 2011; Smith, 2012, p. 10). The repetitive nature of doing science, familiar to any laboratory researcher, is normal science (Jordan & Lynch, 1992). This “repeated trial and error was ‘skill’” acquired by attention and focus, such as hands-on laboratory experiences (Smith, 2012, p. 26). Historical reconstructions, whether of artisan crafts, counting seeds, measuring the boiling point of water, or TMV-inoculation, can be used to address what is perceived to be a reproducibility “crisis” in science. In every instance, historiographic reconstruction has shown the impracticability of exactness in reproducing written work. For us, as scientists and practitioner-historians, we have been excited by how closely our reworking reflects ‘normal science’ by using journal articles, protocol manuals, and in-house experience to plan, perform and evaluate an experiment. Along the way modifications and changes occur, sometimes becoming normalized practice in a laboratory. Scientific manuscripts outline how work was performed, they are narratives of new findings, on the path towards new, even significant, advances in a field of study. If the work is subjected to replication then, by the very nature of scientific practice, somewhat different outcomes may be reported. Hasok Chang, using the term “extension” pushes this point, as we interpret it: Does the historian become a scientist, or considered to be practicing science when the reworking or complementary experiments lead to “something new (though old) about nature” or “genuine original contributions to scientific knowledge”? (Chang, 2011, p. 324). And, vice versa: are scientists who reproduce recently published findings from another laboratory acting as historians of science?

Another aspect of reproducibility is choice. Replicative experiments do not have the scientific prestige of original work, despite the fact that the financial outlay (salaries, reagents, equipment, and publication costs) is equivalent to discovery-based research. Which experiments will be tested for reproducibility? For a specific example, which of 200,000 COVID-19 manuscripts published the past 18 months should be replicated?^24^ As shown by Johan Chu and James Evans, most “scholarly attention” is focused on highly cited papers from well-known labs, making it difficult for “less-established papers—even those with novel, useful, and potentially transformative ideas” to gain attention (Chu & Evans, 2021).

Of papers subjected to replication, how one approaches an experiment is predicated on many parameters, including biased approaches and interpretations.^25^ The influence(r)s guiding the reworking of a particular experiment; interpreting and troubleshooting results; and which findings should be emphasized will differ—even for scientists working together. For example, our reworking of Holmes’, 1934 experiment could be judged unsuccessful: our plants did not look exactly the same, we did not have identical results, and we relied on complementary methods. But, upon reflection, our self-analyses was too harsh. In fact, we learned about the complexity of reworking by localizing temporal and material constraints, identifying decades of changes to “normal” science (training, tools and regents) and making use of advances in virology to understand Holmes’ findings. Importantly, peer-reviewed manuscripts that cited Holmes work, showed us that the LNLs assay, not abscission, remained the standard by which plants were (and are) scored for resistance to TMV.

## Local knowledge and placelessness

In their exploration of allosteric regulation, Angela Creager and Jean-Paul Gaudillière focused on the role of local knowledge and the co-evolution of meaning and experiments within individual locations (Creager & Gaudillière, 1996, p. 90). We interpreted Holmes’ meaning and intent as we reworked his experiment. Robert Kohler has stated that laboratories “are simplified and standardized, stripped of all context and environmental variations; they are places apart from the world—placeless places. It is this odd spatial quality that gives knowledge produced in labs its credibility. The simplicity and sameness of labs helps ensure that experiments turn out the same wherever they are done, which is one of the main reasons why we trust experiment more than other ways of knowing” (Kohler, 2002, p. 191). Yet we did not experience this, and such difficulties in repeating what may be considered normalized science call into question Robert Kohler’s idea of a laboratory as a “placeless place”. As with Creager and Gaudillière’s historiography of allosteric regulation experiments in Berkeley and Paris, we (and Holmes) “worked with different systems, local habits, and distinctive strategies for making decisions” (Creager & Gaudillière, 1996, p. 3). Although we “envisioned” we were “working on the same problems and being part of the same group,” in our case studying TMV, the “decisions made and observations found in each setting affected choices and possibilities” (Creager & Gaudillière, 1996, p. 3). Thus, we had to temper our expectations as we worked to replicate Holmes’ experiments.

In our instance, we were separated not by an ocean, but by time. To perform our experiments we made assumptions about Holmes’ experiments across a gap of decades, yet we “envisioned” ourselves as working on the same problem. Our experiments were informed by Holmes, reconstructing as best we could, his materials and methods. We obtained similar, but not identical results. We learned that location, practice, and a “feeling” for the tools/objects/agents matter greatly when re-working and re-assessing any experiment of the past (Chang, 2011; Creager & Gaudillière, 1996; Keller, 1984; Kohler, 2002).

Again, from Smith, we were reminded that “experiential” or makers’ knowledge is gained by the experimental habit of “doing things over and over” and she (and we) “marveled at the length of time it took to acquire experiential knowledge”(Smith, 2012, pp. 22–23). We found that reworking Holmes’ experiment differed little from initiating a new project including the attendant pitfalls, problem-solving, and interpretation of the results—we had to become wholly immersed in the process of practice. That Holmes intuited the presence of a host resistance gene to TMV infection from an observation of localized necrotic lesion and leaf abscission, shows us a scientist who mastered the craft of working with his research tools, to make foundational advances in virology.

## Footnotes

1 For recent scholarship on the history of tobacco mosaic virus and F. O. Holmes’ contributions to advances in plant virology, see Creager (2002), Creager & Morgan (2008), Scholthof (2004, 2014, 2016), Scholthof & Peterson (2006), Scholthof et al. (1999).

2 The importance of the efforts of the US government and scientists to collect and distribute seed to plant breeders and farmers in the United States has been addressed by Campbell et al., (1999), Curry (2016, 2019), Fitzgerald (1990), Fullilove (2017), Kingsbury (2011), Kloppenburg Jr. (2005), Palladino (1991).

3 For all the successes attributed to plant breeding and genetic resistance to pathogens, a history has yet to be written of the formative years of scientific breeding for plant disease resistance. Textbooks on plant genetics and plant breeding and journals such as *Phytopathology*, *Crop Science*, *Agronomy* and *Genetics* are helpful in understanding the state-of-the art in the early twentieth century to the present. “Plant Pathology: Problems and Progress, 1908–1958” provides an overview of advances in plant pathology (Holton et al., 1959); in this volume, Holmes wrote a commentary on plant virology (Holmes, 1959).

4 On the science and technology of plant breeding and plant genetics in the United States in the early to mid-twentieth century, see Curry (2016), Fitzgerald (1990), Fullilove (2017), Kingsbury (2011).

5 We can speculate that this almost certainly is due to ploidy; that is, the genus *Capsicum* is diploid allowing for straightforward interpretation of species crosses by Mendelian genetics. In contrast, many of the species in *Nicotiana* are polyploid which confounds interpretation of genetic outcomes and introgression of genes of interest. We have addressed the difficulties Holmes had towards introgression of the resistance gene *N* to commercial tobacco from *N. glutinosa* (Scholthof, 2016).

6 By 1959, the USDA *Farmers’ Bulletin* referred growers to Rutgers World Beater No. 13, Yolo Wonder, Keystone Resistant Giant, and Liberty pepper lines, reporting “considerable resistance” to TMV (Boswell et al., 1959, p. 27).

7 In “Doing Biology,” Joel Hagen, Douglas Allchin and Fred Singer used case studies or “historical episodes” of work by individual scientists “that exemplify important characteristics of scientific practice” to “more fully reveal how biology is done” within the context of “science-in-the-making” (Hagen et al., 1997, pp. vi, vii). This book is not a hands-on guide, instead it shows the complexity of science, decision making, and interpreting outcomes with the broad area of the history and sociology of science. Furthermore, common practice(s) also change. What were once standard techniques for plant pathologists are no longer learned in graduate (practicum) courses or the research laboratory. Instead, “hands on” experiences increasingly are replaced with lectures and journal clubs.

8 “TEA” refers to the Transversely Excited Atmospheric Pressure CO_2_ laser, or TEA laser. In Collins’ paper the TEA set refers to the interactions between laboratories in North America and the U.K. who are have developed or are keen to develop the apparatus. Here, Collins discusses networks and networking between labs and how ideas and knowledge travel (Collins, 1974).

9 These recruits “included two plant pathologists, two professors of agronomy, one professor of philosophy (originally trained as a biologist), four biologists, one [human] computer, one practical corn breeder, and one professor and three assistants in plant physiology.” Two of these men by “birth, early life and education” belonged to “the ‘corn belt’ section of the country, and are thoroughly and intimately familiar with maize” and “had experience in corn judging” (Pearl, 1930, pp. 126, 127). We relay this to emphasize the difficulty of reworking experiments; learning the craft behind the experimental technique is key to making and knowing (Smith, 2012).

10 It is important to note that this work on (scientific) material culture is constrained by university rules, and federal and state laws. Laboratory work with viruses and plant materials requires a permit and all lab members be trained in the use of biological materials, chemicals, and (recombinant) pathogens. In this instance, biological materials were used with the approval of the Texas A&M University Institutional Biosafety Committee (Permits IBC2016-130 and IBC2019-139).

11 As reported by Holmes (1934), TMV was maintained on tobacco (*N. tabacum*), as inoculum for the *Capsicum* experiments.

12 The seeds were sown in well-moistened Sungro brand potting mix and transferred to a growth chamber set at ~ 25 °C, 16 h light, 8 h dark ~ 22 °C, 60% humidity, 100–120 μmol/m^2^/s light intensity, watered 3 times/week and fertilized with 20–20-20 (N-P-K) once a week (20 ml/L). Following inoculation, the plants were placed in the laboratory on a plant growth rack at ~ 23 °C 16 h light, 8 h night, and watered 3 times/week without the addition of fertilizer.

13 Of importance to our study, Eric Votava and Paul Bosland reported “‘California Wonder’ should not be included as a standard control in other *Capsicum* research. … The concept of a standard control entails that the ‘control’ can be used in separate labs and in separate experiments and act as a consistent and repeatable benchmark. The dependability of a sample to act as a standard control is cast into doubt if it is shown to contain a high degree of variability or if even a high degree of variability is suspected” (Votava & Bosland, 2002, p. 1102). In 1937, Holmes reported that not all plants of a seed lot responded similarity to infection, suggesting the possibility of a “contaminant of the seed lot” due to inadvertent mixing of seeds (Holmes, 1937, p. 641). Rachel Ankeny and Sabina Leonelli provide an HPS-centered discourse on standard or wildtype organisms (Ankeny & Leonelli, 2021).

14 For example, in the USDA National Germplasm Collection, ~ 15 lines of Tabasco are identified within the *Capsicum frutescens* accessions (https://npgsweb.ars-grin.gov/gringlobal/search). Commercial seed companies then advance lines with specific traits (yield, maturity, fruit color, etc.).

15 The mock-inoculated leaves (control) and TMV-inoculated leaves of Tabasco and bell pepper were photographed several timepoints (days post-inoculation; dpi). Each independent experiment used three susceptible and three resistant plants and the experiments were repeated several times.

16 The infectious cDNA used for these experiments is based on the U1 (common or type) strain (Siegel & Wildman, 1954). This strain, as reported by Milton Zaitlin and H. W. Israel, from “personal recollections of C. A. Knight, W. C. Price and F. O. Holmes suggest that the original isolate used by W. M. Stanley came from J. Johnson of the Univ. of Wisconsin via L. O. Kunkel. The U1 strain (Wittmann & Wittmann-Liebold, 1963) and the German strain ‘vulgare’ (Wittmann-Liebold & Wittmann, 1967) also came from Johnson” (Zaitlin & Israel, 1975).

17 Specifically, we used a binary plasmid containing the coding sequence of TMV with a *gfp* insert (pJL24) (Lindbo, 2007). This plasmid was propagated in *Agrobacterium tumefaciens* strain GV3101, then infiltrated into *N. benthamiana* or *N. tabacum* cv. Turk leaves to initiate the transcription of the recombinant viral genome, TMV-GFP.

18 The California Wonder variety, lacking resistance to TMV, was released in 1937, having first been selected by a California grower in 1928 (Boswell, 1937; Votava & Bosland, 2002). Paul W. Bosland, the New Mexico State University pepper breeder, has documented an exhaustive list of garden catalog descriptions of pepper varieties and the year of commercial release. Bell pepper varieties were bred for TMV resistance by genetic introgression of the Tabasco *L*-gene. Two examples were California Wonder 300 (XP 300) a “thick walled, blocky California Wonder type” released by Asgrow Seed in 1966; and, California Wonder 300 TMR, released in 1999 by Carolina Seeds with “glossy green, thick walled fruit, which turn green to red at maturity, smooth blocky fruit, mostly 4-lobed, averaging 110 × 100 mm fruit size, 72 days to harvest” (Bosland, 2019). https://cucurbitbreeding.wordpress.ncsu.edu/2016/06/03/pepper-a-l/.

19 The 2019 Burpee’s catalog description of Heirloom California Wonder did not indicate TMV resistance.

20 The rapid acceptance of Holmes’ local lesion method and the use of *N. glutinosa* for this bioassay by plant virologists was discussed previously (Scholthof, 2011, 2014).

21 This “reproducibility crisis” is discussed by Marcia McNutt, president of the National Academies of Science and former editor-in-chief of *Science* (McNutt 2014); Jeremy Berg, also a former editor-in-chief of *Science* (Berg 2019); and, a *Nature* collection on “challenges in irreproducible research” at https://www.nature.com/collections/prbfkwmwvz.

22 Angela Creager provides an interesting discussion of the use of laboratory manuals by practitioners (Creager, 2020). This and other articles in the *BJHS Themes* issue “Learning by the Book: Manuals and Handbooks in the History of Science” edited by Elaine Leong, Angela Creager, and Mathias Grote is a particularly helpful volume to grasp the importance of text and its interpretation to perform and interpret science. Peer-reviewed manuscripts should, but mostly do not, include sufficiently detailed materials and methods to allow the work to be reproduced. How-to videos offer an alternative or supplemental format to communicate the use of materials and methods; an example is *JoVE*, a peer-reviewed journal of visual experimentation (jove.com).

23 Similarly, the National Institutes of Health (NIH) has policy and compliance guidelines for “Rigor and Reproducibility” for all grant proposals (https://grants.nih.gov/policy/reproducibility/index.htm). The guidelines, fully implemented in 2020, assert that the “application of rigor ensures robust and unbiased experimental design, methodology, analysis, interpretation, and reporting of results. When a result can reproduced by multiple scientists, it validates the original results and readiness to progress to the next phase of research” (https://www.nih.gov/research-training/rigor-reproducibility). In an extension of this, authors of a recent feature article in *eLife*, a highly ranked journal in the life sciences, suggested the establishment of communities of “'rigor champions' who are committed to promoting rigor and transparency in research” (Koroshetz et al., 2020).

24 Searching “COVID” at PubMed (https://pubmed.ncbi.nlm.nih.gov/?term=covid) yielded 194,281 publications on 15 November 2021. The Web of Science Core Collection (https://clarivate.com) identified 193,280 publications of which ca. 7,764 (4%) are highly cited (accessed 15 November 2021). “Retraction Watch” has recorded 197 retracted or problematic papers (https://retractionwatch.com/retracted-coronavirus-covid-19-papers/; accessed 15 November 2021).

25 A recent report by the National Academies of Science, Engineering and Medicine (2019) addressed these topics in *Reproducibility and Replicability in Science*, with a particular focus on replication in chapter 7.
